# Global epidemiology of telogen effluvium after the COVID-19 pandemic: A systematic review and modeling study

**DOI:** 10.1016/j.jdin.2024.08.018

**Published:** 2024-09-24

**Authors:** Jae Joon Jeon, You Hyun Kim, Hyunjun Kang, Min Chul Ha, Seung-Won Jung, Hyunsoo Son, Myung Ha Kim, Won-Soo Lee, Solam Lee

**Affiliations:** a Department of Dermatology, Yonsei University Wonju College of Medicine, Wonju, Republic of Korea; b Department of Medicine, Yonsei University Wonju College of Medicine, Wonju, Republic of Korea; c Department of Bioengineering, College of Engineering, University of Washington, Seattle, Washington; d Yonsei Wonju Medical Library, Yonsei University Wonju College of Medicine, Wonju, Republic of Korea

**Keywords:** COVID-19, epidemiology, ethnicities, hair loss, meta-analysis, minorities, prevalence, skin of color, telogen effluvium

*To the Editor:* Telogen effluvium (TE) is a nonscarring alopecia characterized by diffuse hair loss, with various etiologies such as pregnancy, malnutrition, and infection. A recent US study reported a three-fold increased incidence of TE during the coronavirus disease 2019 (COVID-19) pandemic.^1^ During this period, patients with hair loss increased to nearly 10% of all dermatology outpatient visits.^2^ However, standardized evidence of the worldwide TE prevalence is insufficient. Therefore, this study aimed to investigate the global, regional, and national epidemiology of TE.

We searched publications from six databases from inception up to March 6, 2024 (Supplementary Table I and Fig 1, available via Mendeley at https://doi.org/10.17632/ccy9yhh36m.1). We included cross-sectional and cohort studies that could calculate prevalence rates by country, classified according to the Global Burden of Disease classification (Supplementary Table II, available via Mendeley at https://doi.org/10.17632/ccy9yhh36m.1). To address heterogeneity, we stratified the data by diagnostic method (self-reporting or physician-/dermatologist-diagnosed) and study setting (before or after the pandemic). The risk of bias was evaluated using the Joanna Briggs Institute Critical Appraisal Checklist (Supplementary Table III, available via Mendeley at https://doi.org/10.17632/ccy9yhh36m.1). We used a Bayesian hierarchical linear mixed model to estimate the prevalence of TE with 95% credible intervals (CrIs, details in Supplementary Text and Fig 2, available via Mendeley at https://doi.org/10.17632/ccy9yhh36m.1). This method was utilized for estimating prevalence of diverse dermatologic diseases from our previous works.^3^^,^^4^ This review was registered in PROSPERO (CRD42024517571).

We included 90 studies from 26 countries, covering 61.9% (13/21) of total Global Burden of Disease regions (Supplementary Tables IV and V, and Fig 3, available via Mendeley at https://doi.org/10.17632/ccy9yhh36m.1). Of these, 27 (30%) were considered to report the prevalence of TE after the pandemic (Supplementary Table VI, available via Mendeley at https://doi.org/10.17632/ccy9yhh36m.1). The global prevalence of TE was estimated at 5.41% (95% CrI 2.73% to 11.22%) after the pandemic (Supplementary Table VII, available via Mendeley at https://doi.org/10.17632/ccy9yhh36m.1, Figs 1 and 2), which was considerably greater than 3.44% (95% CrI 1.96% to 6.28%) before the pandemic (Supplementary Table VIII, and Figs 4 and 5, available via Mendeley at https://doi.org/10.17632/ccy9yhh36m.1). The prevalence estimates by country were lowest in the US (2.08%; 95% CrI 1.08% to 4.15%) and highest in China (13.33%; 95% CrI 4.03% to 51.04%) after the pandemic. At the regional level, the estimated TE prevalence after the pandemic varied, ranging from 2.83% (95% CrI 1.23% to 6.94%) in high-income North America to 11.11% (95% CrI 3.42% to 44.33%) in East Asia. In addition, most studies reporting prevalence showed a much higher female predilection.

**Fig 1:**
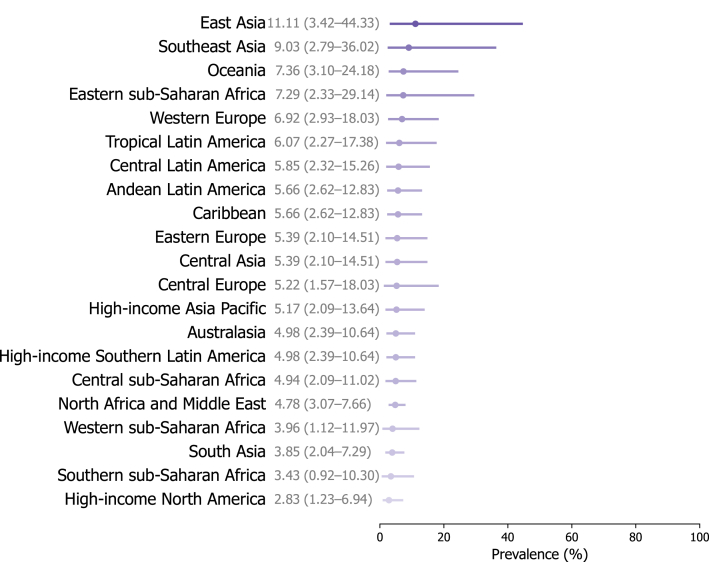
The estimated lifetime prevalence rates of telogen effluvium after the COVID-19 pandemic according to world regions. The numbers and error bars present the estimated prevalence with 95% credible intervals (CrIs).

**Fig 2:**
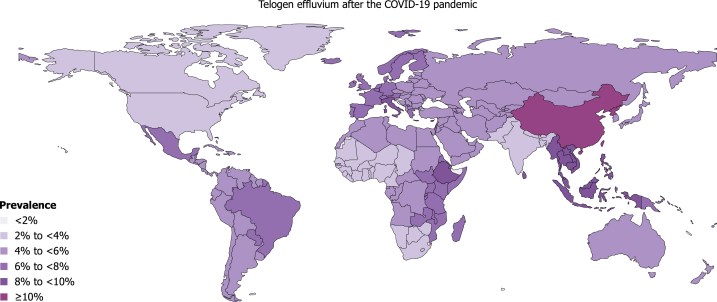
Physician-diagnosed or dermatologist-diagnosed prevalence of telogen effluvium after the COVID-19 pandemic. Details about the estimated prevalence of telogen effluvium after the pandemic by country, region, and super-region are given in Supplementary Table VII.

Our modeling study provides an up-to-date epidemiology of TE before and after the COVID-19 pandemic. Damage to hair follicles following a systemic inflammatory response may lead to TE; however, the mechanism by which COVID-19 induces TE remains unclear. The incidence of TE among Hispanic/Latinx and Asians significantly increased during the pandemic.^1^ Similarly, our study showed generally higher TE prevalence estimates in Asia and Latin regions irrespective of the pandemic. Considering the increasing proportion of skin-of-color populations in the US, further studies on the predisposition of TE and other hair disorders according to race and ethnicity are necessary.^5^

Our study had some limitations. First, most of the included studies were conducted on outpatient populations. Second, the data were insufficient to analyze acute and chronic TE separately. Nevertheless, we examined the global impact of COVID-19 on TE diagnoses. Our findings provide comprehensive insights into the regional distribution of TE.

## Conflicts of interest

None declared.
